# Efficacy of a Novel Computerized Aid in Designing Removable Partial Dentures

**DOI:** 10.7759/cureus.54581

**Published:** 2024-02-20

**Authors:** Rajmohan Sivamani Chidambaram, Sudha Rajmohan, Sivakumar Manickam, Rachappa Mallikarjuna, Triveni Nalawade, Sanjay Saraf

**Affiliations:** 1 Department of Prosthodontics, Oman Dental College, Muscat, OMN; 2 Department of Preclinical Conservative Dentistry, Oman Dental College, Muscat, OMN; 3 Department of E-learning, Oman Dental College, Muscat, OMN; 4 Department of Pedodontics and Preventive Dentistry, Oman Dental College, Muscat, OMN; 5 Department of Oral Biology, Oman Dental College, Muscat, OMN

**Keywords:** self-directive learning, online interactive application, removable partial denture design, computer-aided learning, e-learning

## Abstract

Background: The development of computerized aids for dental education offers potential benefits in teaching complex procedures, such as the design of removable partial dentures (RPDs). This study aimed to assess the efficacy of a novel computerized tool in enhancing the ability of both dental students and practicing dentists to design RPDs, as well as to evaluate its utility as an interactive educational instrument.

Methods: A cohort comprising a total of 75 individuals (25 practicing dentists and 50 undergraduate dental students) was enlisted. Participants were introduced to an online interactive application tailored for the design of RPDs. They were tasked with resolving clinical scenarios that necessitated the formulation of an RPD. Throughout the exercise, users were provided with hints addressing errors made during the process, fostering self-directed learning for improved RPD design. Post-interaction, the perceptions of both dentists and students regarding the tool were gauged through a comprehensive questionnaire.

Results: The deployment of the online interactive application demonstrated significant promise in the effective design of RPDs, facilitated by self-directed learning. It also appeared to enhance the proficiency of practicing dentists in formulating partial dentures.

Conclusion: The computerized aid evaluated in this study provided an effective platform for both dental education and practice. It not only supported self-directed learning in the design of RPDs but also improved the efficiency of professional dentists in their clinical design work.

## Introduction

An important part of learning about prosthodontics before applying what you have learned is learning about how to make removable partial dentures (RPDs). This discipline is historically recognized for its intricate nature and the consequent challenges it poses to learners. To mitigate these educational barriers, the integration of autonomous learning strategies alongside conventional pedagogical methods has been proposed.

Autodidactic activities such as independent literature reviews, collaborative academic dialogues, critical self-reflection, and digital learning platforms are increasingly embraced within educational frameworks. The advent of internet-enabled electronic devices has particularly propelled e-learning to the forefront of self-instructional methods. E-learning's compatibility with traditional teaching paradigms [1] and facilitation of a learner-controlled pace [2] have been documented. An investigation by Lechner et al. [3] reported the substitution of conventional lectures with e-learning modules in RPD design, resulting in superior academic outcomes.

Despite the availability of numerous software applications that delineate RPD design processes [4-9], their limited interactivity has been identified as a contributing factor to diminished student engagement and a subsequent decline in the efficacy of self-directed learning. Contemporary educational demands call for platforms that are not only pedagogically supportive but also user-friendly, with longevity extending into postgraduate professional practice.

In response to this educational imperative, an initiative was undertaken to develop an interactive online application at the Oman Dental College in Muscat, Oman. This application is designed to be interactive, responsive, and adaptive, with characteristics that are hypothesized to enrich the learning experience. The interactivity of the tool provides immediate engagement with the user, fostering an accelerated learning curve. Its responsive design ensures accessibility across various digital devices, including laptops, smartphones, and tablets, thereby offering learners an unparalleled degree of flexibility. The adaptive feedback mechanism of the software is tailored to guide learners effectively through the nuances of RPD design. The objective of this investigation, therefore, was to critically assess the capacity of both dental practitioners and undergraduates to utilize this novel e-learning application, as well as to evaluate their perceptions of its educational value.

## Materials and methods

Study design

The study incorporated a total of 75 individuals, of whom 50 were undergraduate dental students who had recently concluded their preclinical training in the domain of RPD design, and the rest 25 were dental professionals, each with a certain amount of experience and knowledge in coming up with and using RPDs for simple clinical cases. They stood on the threshold of transitioning to clinical patient management. Prior to this study, their foundational knowledge was constructed through traditional pedagogical strategies comprising lectures, tutorials, and laboratory exercises tailored to preclinical skill development.

This new computerized tool was created in-house, which makes it cheaper than traditional ways of making RPDs. Moreover, adding interactive modules also makes it easier for people to understand and picture complicated design ideas, which helps them better remember and execute complex design principles.

Pedagogical framework

A pioneering computer-assisted e-learning module was conceived, employing a logic flow reminiscent of traditional RPD design methodology. A decisional tree architecture underpinned the program's logic, guiding users through an algorithmic sequence of choices culminating in clinically sound RPD designs. The system was programmed to recognize several valid outcomes as a product of the decision-making process.

Collaborative development team

The e-learning tool was developed at Oman Dental College, with contributions from (1) senior prosthodontics faculty, responsible for formulating the sequential decision-making logic, (2) a software engineer tasked with translating the pedagogical framework into an operational algorithm, and (3) a web development specialist is charged with crafting the interface design and user navigation commands, along with integrating tracking and reporting functions. This tool has not been published yet.

Technological framework

The RPD design e-learning software was engineered utilizing Hypertext Markup Language 5 (HTML5) for its web-based infrastructure and Action Script 2 for the implementation of the decision tree's interactive components. This combination facilitated the creation of a dynamic educational experience within the virtual learning environment (Moodle 2.7+).

Clinical scenario presentation

**Figure 1:**
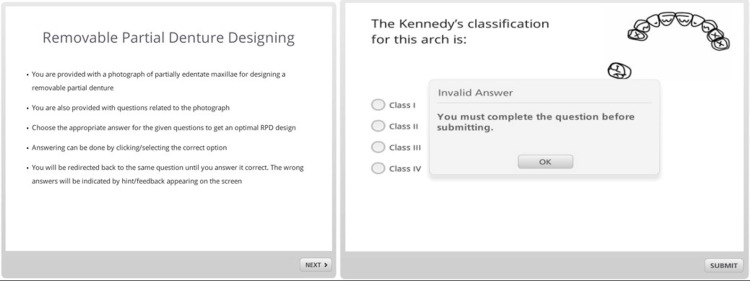
Clinical scenario depiction

Participants were presented with a standardized clinical scenario (visualized in Figure 1) and instructed to utilize the online interactive e-learning module to formulate an RPD design. The participants were further asked to provide their respective comments and experiences pertaining to the utilization of the computerized aid in the design process.

Prosthodontic design process

The methodology required participants to (1) analyze the patient's maxillary arch [10,11], (2) interact with a digitally animated dental cast depicted from multiple orientations, including various tilts to underscore the concept of RPD retention, (3) select an appropriate major connector for the RPD, (4) identify and select potential abutment teeth for clasp placement, (5) design the clasps for the selected abutment teeth, and (6) choose suitable materials for the construction of the clasps.

Decision validation and visual feedback

**Figure 2:**
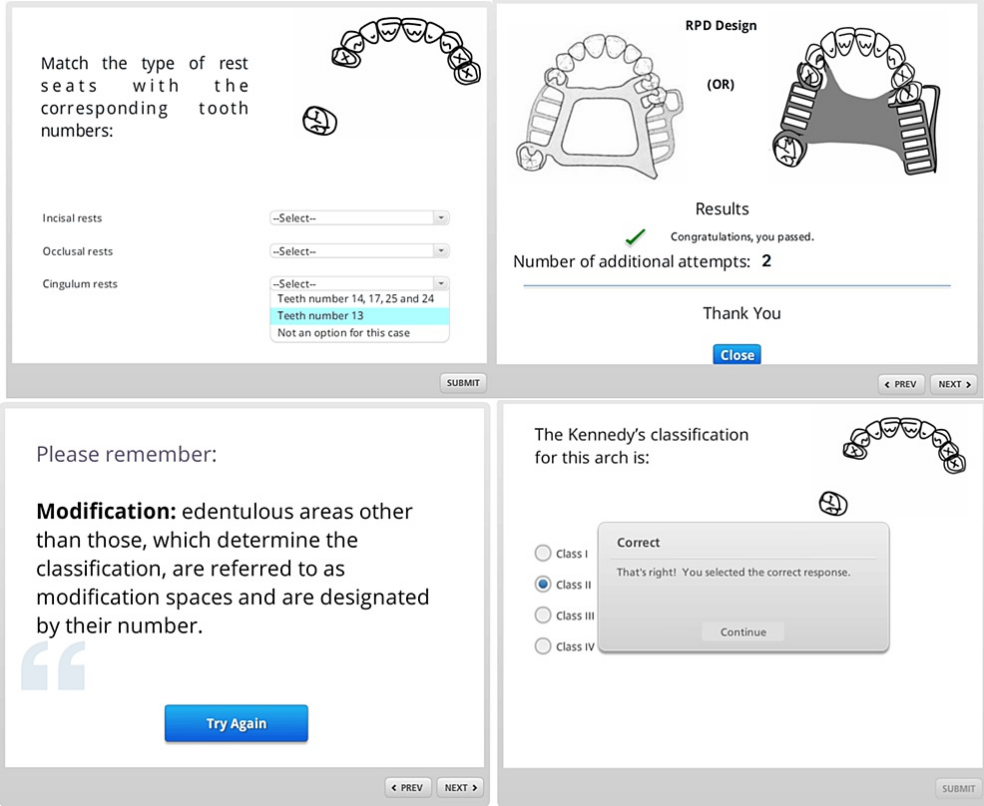
Depiction of different visual cues and depiction of the final RPD design RPD: Removable partial denture

The e-learning tool's instructional design required participants to make a series of clinically acceptable choices, with immediate feedback provided for any unapproved selections. Progress through the module was contingent upon correct decision-making, as indicated in Figures 1, 2. Visual cues, detailed in Figure 2, were employed to guide participants through the selection process, ensuring adherence to prosthodontic principles. Upon completion, an animated representation of the RPD design was displayed for review (Figure 2).

Interaction tracking and formative assessment

Integrated tracking tools within the application captured key data such as time spent, number of attempts, and scores, allowing for a comprehensive analysis of the tool's effectiveness. This formative assessment approach aims to enhance learning outcomes and refine the educational process.

Post-interaction feedback

Participants' perceptions were evaluated through a post-interaction questionnaire, focusing on their comfort level with the application and its utility in both learning and clinical practice. The anonymously collected responses were intended to direct future improvements to the e-learning module, ensuring it met educational requirements and facilitated self-directed learning effectively.

Statistical analysis

We used the chi-square test of Independence to examine the relationship between the participant group (students and dentists) and their responses on efficiency, technical queries, technical competency, affordance, and RPD design preference. For categories with low sample sizes, such as "no difference" in efficiency and "not comfortable" with affordance, Fisher's exact test was used to ensure accurate analysis.

Ethical considerations

Due consent was obtained from all the participants who were a part of this study, and the Institutional Review Board number “ODC/2022/167” was assigned to this investigation by the Ethical Review Board of Oman Dental College.

## Results

The participants' perceptions of the tool's different aspects that were measured are depicted in Table 1.

**Table 1:** Different observations of the participant's responses (n%) RPD: Removable partial denture

Observation	Students' response percentage	Dentists' response percentage
Tool efficiency		
Better	83%	71%
Low	12%	20%
No difference	5%	9%
Technical queries of the tool		
Satisfied	81%	83%
Not satisfied	19%	17%
RPD designing competency		
Sufficient	61%	73%
Not sufficient	24%	12%
Need to brush up	15%	15%
Affordance		
Comfortable	68%	61%
Average	27%	29%
Not comfortable	5%	10%
Preferred method		
Computer-based	63%	55%
Conventional	27%	35%
Combined	10%	10%

The data revealed that a significant majority of the participants, comprising 83% of students and 71% of dentists, acknowledged the tool's efficiency in RPD design. Conversely, a minority of students (12%) and dentists (20%) reported the tool's low efficiency, while only 5% of students and 9% of dentists observed no difference in efficiency compared to alternative methods.

Regarding technical queries, there was no gap between students and dentists concerning the online application's technical aspects. About 81% of the students and 83% of the dentists were satisfied with the technical aspects.

Regarding the competency of designing the RPD, there was a discernible gap between students and dentists. Sixty-one percent of the students expressed competency, which contrasted with the 83% of dentists who were competent. while an equal proportion of 15% in each group indicated the need for improvement in their design skills. Notably, a higher percentage of students (24%) lacked sufficient competency in comparison to dentists (12%), potentially reflecting the dentists' greater experience in the design.

The majority of students (68%) and dentists (61%) reported comfort in using the application. A smaller segment of students (27%) and dentists (29%) deemed the application's usability average. Only a minimal fraction of students (5%) found the application uncomfortable, whereas dentists (10%) reported discomfort. The data also highlighted that 61% of dentists rated the application as comfortable, 29% as average, and 10% as not comfortable, reinforcing the notion that the majority of both demographic groups rated the application as user-friendly.

Involving 50 students and 25 dentists, the study found that the majority of students, 63%, favored the computer-aided design (CAD) method. In contrast, only 27% of students preferred the conventional method, with the remaining 10% opting for a combination of both methods. On the other hand, the dentists' preferences were more varied. A slight majority of 55% chose the CAD method, whereas a significant 35% still preferred conventional methods. A small proportion of dentists, 10%, showed a preference for the combined approach. Despite the varied preferences, the CAD method emerged as the most popular among both students and dentists. However, when considering the collective preferences of the 50 students and 25 dentists, the data revealed a divergence between the two groups. A majority of dentists, 55%, showed a preference for the conventional method, which differed from the students' inclinations, with 63% favoring the CAD method. The combined method was the second choice for students at 27%, equating to the conventional method.

The other findings indicate a differential response to digital design interfaces between novice and experienced practitioners. The majority of students identified the CAD system as superior in terms of design efficiency and time management compared to traditional methods. Conversely, experienced dentists not only affirmed the efficiency of the CAD system but also acknowledged its utility as a refresher for theoretical competencies that had diminished post-graduation. Outliers in the dataset comprised two student respondents who reported a preference for traditional educational modalities over the e-learning application, citing greater comfort with conventional instructional techniques. The chi-square test of independence results (Table 2) indicated that there were no statistically significant differences between students and dentists in terms of efficiency, technical queries, technical competency, affordance, and preferred method for RPD design, all above the conventional alpha level of 0.05. Specifically, for the efficiency category, while there appeared to be a trend where a higher percentage of students (83%) than dentists (71%) found the computer-based method to be better, the difference was not statistically significant (p=0.075). Similar non-significant results were observed in technical query satisfaction (p=0.570), technical competency (p=0.160), affordance (p=0.389), and preferred method (p=0.296).

**Table 2:** Chi-square analysis observations

Observation	Students (n)	Dentists (n)	Chi-square value	Degrees of freedom	p-value
Efficiency					
Better	41.5	17.75	5.18	2	0.075
Low	6	5			
No difference	2.5	2.25			
Technical queries					
Satisfied	40.5	20.75	0.32	1	0.570
Not satisfied	9.5	4.25			
Technical competency					
Sufficient	30.5	18.25	3.67	2	0.160
Not sufficient	12	3			
Need to brush up	7.5	3.75			
Affordability					
Comfortable	30.5	17	1.89	2	0.389
Average	14.5	6.75			
Not comfortable	5	1.25			
Preferred method					
Computer-based	27.5	15.75	2.43	2	0.296
Conventional	17.5	6.75			
Combined	5	2.5			

Fisher's exact test (Table 3) was employed for the categories with small expected counts, which in this dataset were "no difference" in efficiency and "not comfortable" in affordance. The results showed that differences in these specific categories were also not statistically significant, with p-values of 1.000 and 0.482, respectively. This suggests that there was no strong evidence of a difference in the proportions of students and dentists who reported "no difference" in efficiency or felt "not comfortable" with the RPD design using the computerized aid.

**Table 3:** Fisher’s test observations

Observation	Students	Dentists	Fisher's exact test p-value
Efficiency			
Better	41.5	17.75	1.000*
Low	6	5	
No difference	2.5	2.25	
Affordance			
Comfortable	30.5	17	0.482*
Average	14.5	6.75	
Not comfortable	5	1.25	

## Discussion

The majority of the participants in this study gave positive feedback on how well it worked as a teaching tool. Quantitative evaluations showed a widespread appreciation for the application among both student and professional cohorts, with a particular focus on the engagement that the e-learning model provided. Adding interactive modules made it much easier for people to understand and visualize complicated design concepts, which improved their ability to mentally absorb RPD design principles. The pedagogical advantage of such interactive systems lies in their capacity to present theoretical concepts in a dynamic and accessible manner, thereby augmenting the learning curve [12].

The application's feedback mechanisms, which are crucial in promoting self-guided learning, further increase its pedagogical efficacy. These mechanisms enable educators to fine-tune the educational material to the evolving requirements and proficiency levels of learners [13]. Moreover, the bidirectional feedback between educators and students and among peers is instrumental in the learning process; however, the effectiveness of this interaction is contingent upon the responsiveness and digital demeanor of the instructors [14].

Foster et al. [15] studied how dental students responded to a computer-based treatment planning tool. Their study shows how these kinds of tools can help improve clinical cognitive skills by giving students a collection of patient cases with varying levels of difficulty. The accessibility of this virtual repository for after-hours study is posited as a critical feature for an effective educational tool, underscoring the need for versatility in instructional design.

The study also acknowledges the value of tactile experience in RPD design, suggesting that while e-learning offers substantial instructional benefits, it should complement rather than replace hands-on techniques. The structured nature of the program is noted to aid in the logical progression of treatment planning. The e-learning system, with its diverse array of functionalities and adaptable framework, demonstrated potential for transforming pedagogical methodologies and the educational milieu within dental academia. Despite the numerous benefits associated with the e-course, its utility for monitoring individual student progression was not optimally leveraged.

Conceição et al. [16] performed a comparative analysis of the digital techniques used in the fabrication of RPDs. The findings indicate a predominant preference for extra-oral scanning over intra-oral scanning modalities. Among the digital methodologies assessed, CAD emerged as the most frequently employed technique for the conceptualization and planning of RPD structures. The review by Mai et al. [17] highlighted a gap in the literature pertaining to the precision of RPD frameworks fabricated through milling techniques. The meta-analysis showed that there was a statistically significant difference between the fit of frameworks made using CAD/CAM methods and the traditional method of lost wax and casting.

In contrast to our findings, Chen et al. [18] conducted a study with a technical emphasis on optimizing RPD design through computational methods and additive manufacturing, aiming to improve the uniformity of contact pressure on the mucosa and minimize associated clinical complications. They report a significant reduction in peak contact pressure and an increase in uniformity following their optimization process, leveraging 3D printing technology to prototype the optimized dentures. Kalberer et al. [19] contribute to the conversation by comparing the trueness of complete dentures produced by two different CAD/CAM processes: milling and 3D printing. Their in-vitro study reveals that milled dentures achieve significantly better trueness than those that are 3D printed, suggesting a potential preference for milling in situations where precision is paramount. While the primary study gauges subjective responses to the efficiency and usability of CAD tools in dental education and practice, Chen et al. [18] and Kalberer et al. [19] provide objective evidence related to the technical performance of CAD/CAM-fabricated dentures.

A number of other studies in this regard have observed that e-learning tools, not just across the prosthodontic domain but other facets of dentistry as well, have had a positive effect in terms of enhancing student engagement. Spalthoff et al. [20] observed that standardized virtual dental arches were practically adequate because virtual reconstruction of every edentulous case using these virtual arches was possible without any additional modifications, and that it was also possible to fabricate clinically acceptable temporary prostheses using a comprehensive digital workflow based on standardized digital dental arches. Dobrzański et al. [21], through their review, elucidated the concept of "Dentistry 4.0," where they explain the importance and future significance of CAD/CAM and machine learning tools within prosthodontics. However, they deviate from one of the primary objectives of our study since they did not elaborate upon these tools' roles in the learning process.

Bansod et al. [22] in their original research revealed the substantial challenges that dental students and instructors confront when it comes to e-learning in prosthodontics which could be used to advise the university's dentistry education section in developing an effective and productive online teaching platform aimed at improving students' learning experiences, an observation that we share through our findings as well. E-learning was further successfully used in a dental school's curriculum to enhance students' perceptions of fundamental concepts and enabled students to apply that knowledge to clinical cases in the study done by Turkyilmaz et al. [23].

The study, though comprehensive in its approach, did encounter several limitations that must be acknowledged when interpreting the findings. From a design and pedagogical standpoint, the e-learning module was built completely in-house upon a decisional tree architecture by an expert from within the institution, which, while innovative, may not fully encapsulate the complexity and nuance of RPD design encountered in real-world clinical practice. The reliance on algorithmic pathways may inadvertently limit the scope of design scenarios and overlook less common but clinically relevant situations. Technologically, the use of HTML5 and ActionScript 2 provided a robust and interactive platform, but the technological framework of the e-learning tool might not be compatible with all devices or web browsers, which could impede access and the user experience. The clinical scenarios presented to participants were standardized, which, while beneficial for consistency and comparison, may not fully reflect the variety of challenges faced in actual clinical settings. Furthermore, the design process for this aid was highly structured, requiring participants to follow a series of predetermined steps. Some may not appreciate the iterative and dynamic nature of design in clinical practice. While the e-learning tool provided immediate feedback for unapproved selections, this mechanism may not effectively mimic the complex decision-making process and the subtleties of clinical judgment required in practice. The feedback might also reinforce a single correct pathway, potentially discouraging exploration and the development of individualized problem-solving skills.

## Conclusions

The results of this study concluded that e-learning applications can be used effectively as a self-directed learning tool along with traditional lecture courses in RPD design. It helps in solving the problem using analytical skills and applying factual knowledge to real-life patient management. Care must be taken while integrating e-learning into the curriculum to ensure sufficient resources are available for computer access; however, in today’s world, it is unusual to find a student or graduated dentist who doesn’t own a smartphone or tablet. It is also important to ensure that alternative resources are also made available for those who see technology as a possible barrier to learning. The sound and well-versed technological knowledge of today’s students make the capability of understanding the principles of the so-called traditionally difficult RPD design easy with this self-directed online e-learning application. They can design clinically acceptable RPDs using a blended approach of e-learning and traditional methods. The project has shown that the students not only enjoyed it but also appreciated the opportunity to do so.
